# Artificial intelligence to automate assessment of ocular and periocular measurements

**DOI:** 10.1177/11206721241249773

**Published:** 2024-05-06

**Authors:** Khizar Rana, Mark Beecher, Carmelo Caltabiano, Carmelo Macri, Yang Zhao, Johan Verjans, Dinesh Selva

**Affiliations:** 1 Department of Ophthalmology & Visual Sciences, South Australian Institute of Ophthalmology, University of Adelaide, North Terrace, SA 5000, Australia; 2 Australian Institute for Machine Learning, 1066 The University of Adelaide, SA 5000, Adelaide, Australia

**Keywords:** Periocular, machine learning, marginal reflex distance

## Abstract

**Purpose:**

To develop and validate a deep learning facial landmark detection network to automate the assessment of periocular anthropometric measurements.

**Methods:**

Patients presenting to the ophthalmology clinic were prospectively enrolled and had their images taken using a standardised protocol. Facial landmarks were segmented on the images to enable calculation of marginal reflex distance (MRD) 1 and 2, palpebral fissure height (PFH), inner intercanthal distance (IICD), outer intercanthal distance (OICD), interpupillary distance (IPD) and horizontal palpebral aperture (HPA). These manual segmentations were used to train a machine learning algorithm to automatically detect facial landmarks and calculate these measurements. The main outcomes were the mean absolute error and intraclass correlation coefficient.

**Results:**

A total of 958 eyes from 479 participants were included. The testing set consisted of 290 eyes from 145 patients. The AI algorithm demonstrated close agreement with human measurements, with mean absolute errors ranging from 0.22 mm for IPD to 0.88 mm for IICD. The intraclass correlation coefficients indicated excellent reliability (ICC > 0.90) for MRD1, MRD2, PFH, OICD, IICD, and IPD, while HPA showed good reliability (ICC 0.84). The landmark detection model was highly accurate and achieved a mean error rate of 0.51% and failure rate at 0.1 of 0%.

**Conclusion:**

The automated facial landmark detection network provided accurate and reliable periocular measurements. This may help increase the objectivity of periocular measurements in the clinic and may facilitate remote assessment of patients with tele-health.

## Introduction

Accurate and reliable periocular structures is important in diagnosing and monitoring various ophthalmological conditions. Eyelid position can be altered in orbital diseases like thyroid eye disease, tumours, and trauma. Presently, manual measurement methods, such as marginal reflex distance (MRD) 1 and 2, are employed to determine the vertical distance from the corneal light reflex to the upper and lower eyelid margins, respectively. However, these manual approaches are subjective, dependent on the operator's skill, and often demand a level of patient cooperation, posing challenges in certain patient groups like children and cognitively impaired adults. Additionally, with the increasing adoption of telehealth, physical execution of such measurements may become impractical.

We sought to develop a deep learning model for facial landmark detection to automatically detect periocular landmarks and conduct accurate periorbital and eyelid measurements.

## Methods

We prospectively enrolled participants presenting to the Royal Adelaide Hospital ophthalmology clinic who were 18 years of age or older and gave written informed consent. Patients with ocular misalignment, pupil abnormalities, or corneal pathology affecting the light reflex were excluded from the study. The institutional human research ethics committee approved the study. Study procedures adhered to the principles of the Declaration of Helsinki.

### Image collection

In a well-lit room, seated participants were placed 1 metre from a Nikon D90 camera equipped with a 60 mm lens and positioned on a stand at eye level. Participants were asked to look straight, and the photographs were taken head-on. To enable accurate calibration, a circular green adhesive dot sticker with a diameter of 24 mm was placed on the subject's forehead, allowing for the conversion of pixels to millimetres.

### Image analysis

**Figure 1.**
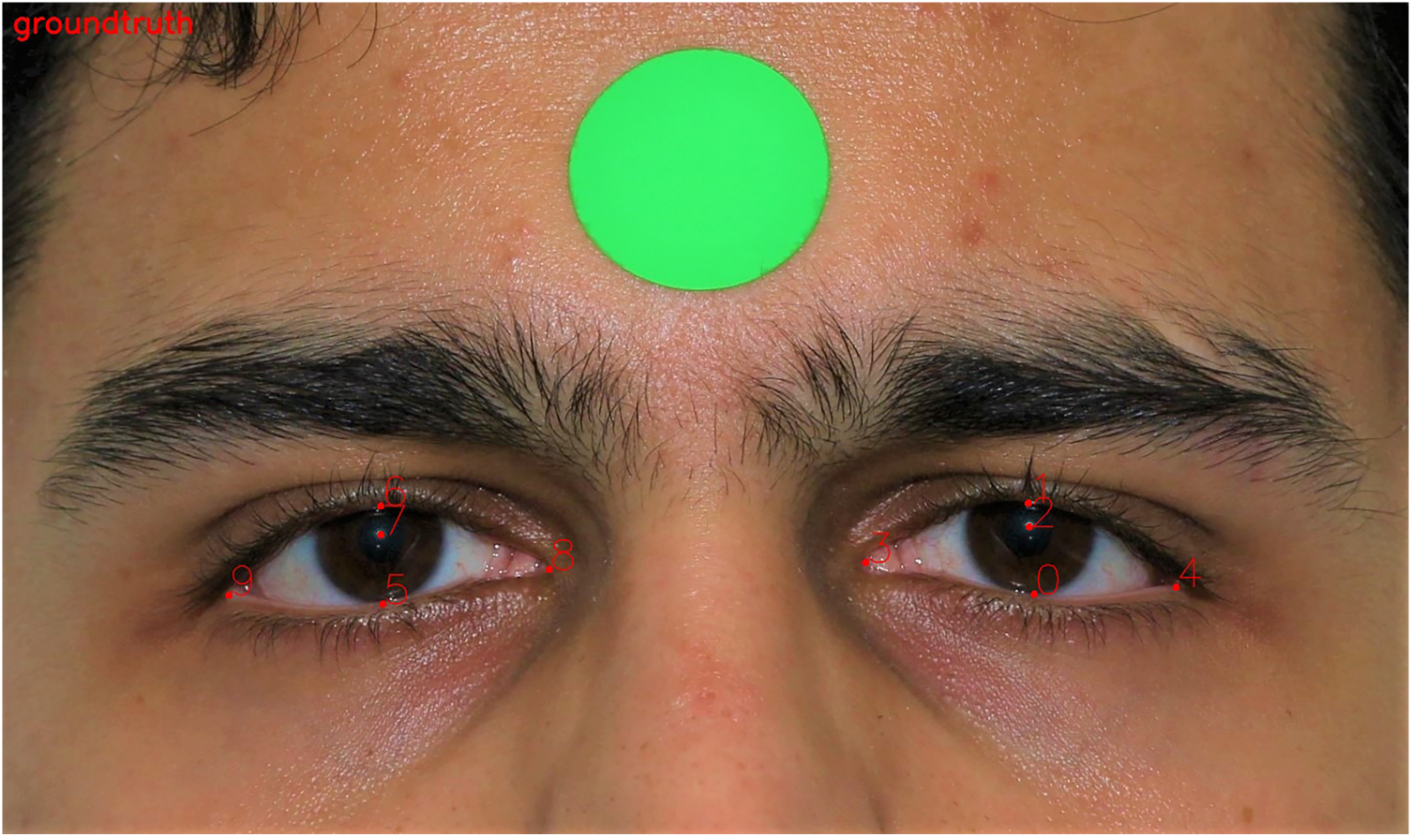
The periocular landmarks were manually segmented.

**Figure 2.**
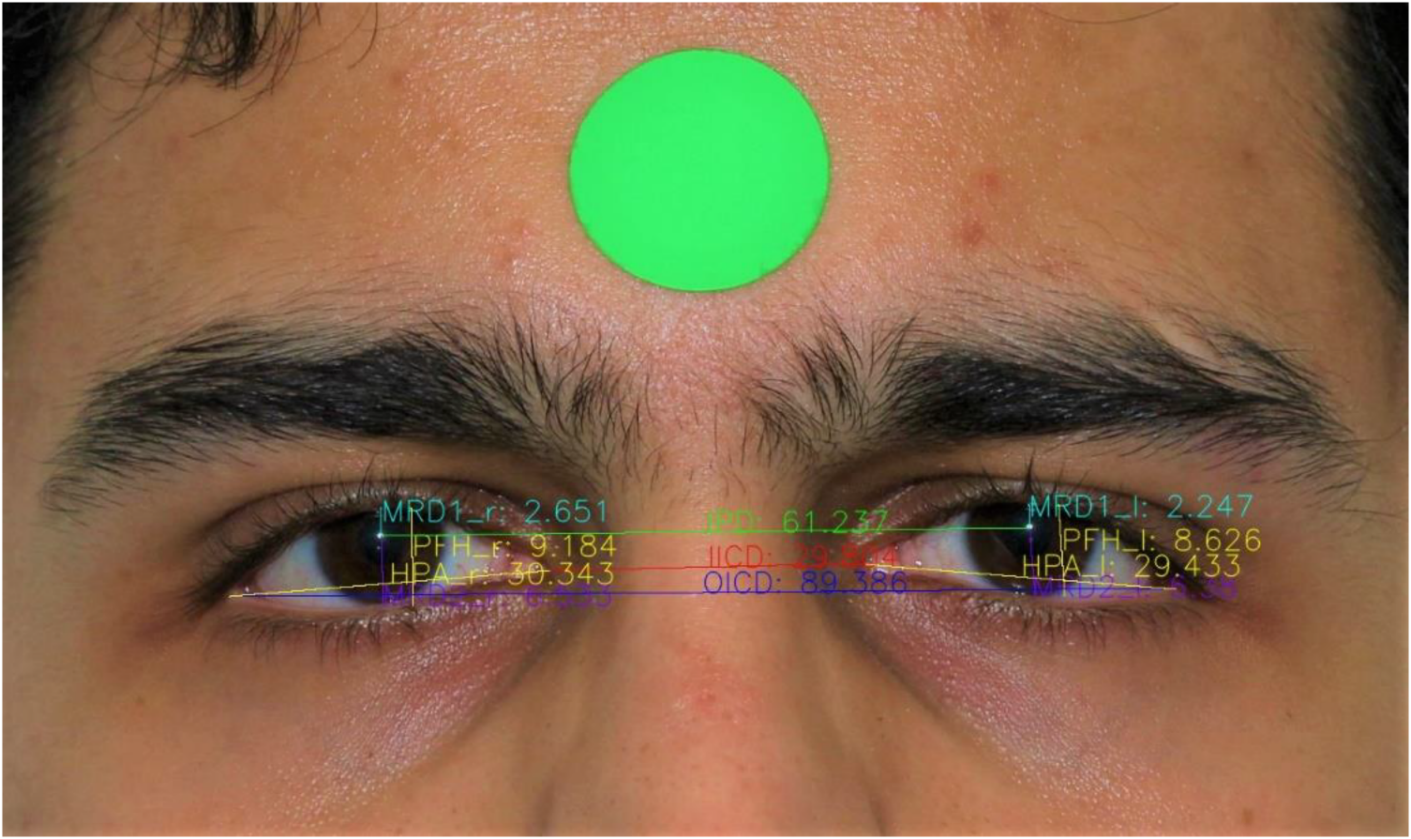
The periocular landmarks were used to calculate the periocular dimensions.

The images were upload onto Labelbox, a popular web-based annotation tool for segmentation and classification systems.^1^ Ten periocular landmarks, including the pupillary centre, the midline of the upper eyelid margin, the midline of the lower eyelid margin, medial canthi, and lateral canthi for each eye were manually annotated (Figure 1). The distances between the periocular landmarks were computed using the open-source OpenCV library. The calculated dimensions included MRD1, the vertical distance from the pupillary centre to the centre of the upper eyelid margin; MRD2, the vertical distance from the pupillary centre to the centre of the lower eyelid margin; palpebral fissure, the vertical height between the upper and lower eyelids, derived by summing MRD1 and MRD2; inner intercanthal distance (IICD), the horizontal distance between the medial canthi; outer intercanthal distance (OICD), the horizontal distance between the lateral canthi; interpupillary distance (IPD), the horizontal distance between the centres of the two pupils; and horizontal palpebral aperture (HPA), the horizontal distance between the medial and lateral canthi within one eye (Figure 2).

### Deep learning model development

To make our framework reproducible, we adopted a widely used backbone network HRNet-v2 as our landmark detection model to predict designed facial landmarks.^2^ HRNet-v2combines the representations from all the high-to-low resolution parallel streams. Specifically, the input of the landmark detection model HRNet-v2 is a facial image of size w×h , and the output of the landmark detection model is likelihood heatmaps $H\;=\{H_{l}{\}}_{l=1}^{L}$ for *L* pre-defined facial landmarks. In the design of HRNet-v2, the size of the output heatmaps is reduced by four times. As described in Section of image analysis, *L* is equal to ten for the landmark detection model. To optimise the landmark detection model, we employ the Mean Squared Error (MSE) loss function to compare the predicted heatmaps H and the ground-truth heatmaps H* , which are generated from the annotated 2D facial landmarks. Here the values on the heatmap H are computed from a 2D Gaussian distribution centred at landmark *l*. Therefore, the loss function between the predicted heatmaps H and ground-truth heatmaps H* is defined as $Loss(H,H*)=||H-H*|{|}^{2}$ . When the training is finished, the landmark coordinates can be generated from the corresponding prediction H* by finding the coordinates of the highest heatmap value and up sampling back the original image size.

The complete dataset was randomly split into training (70%) and evaluation (30%) sets. Each image in the proposed dataset had 10 manually annotated landmarks, as shown in Fig. 1. All the face images including both training and testing images were scaled to 512 × 256. Our landmark detection model was trained using Pytorch version 1.7.0 on a single NVidia RTX 3090 GPU with 24GB video memory. Data augmentation was performed to improve the robustness of the network to data variations. Image augmentation was performed by in-plane rotation (± 30 degrees), scaling (0.75–1.25) and random horizontal flips (probability 50%). The Adam optimizer was used with a mini-batch size of 16 for 60 epochs. The base learning rate was 10^−3^ and decayed to 10^−4^ at the 30th epochs and 10^−5^ at the 50th epochs respectively.

### Statistical analysis

Human and AI predicted measurements were summarised by mean and standard deviation. Agreement between human measurements and AI predicted measurements was assessed using Bland-Altman plots with 95% confidence intervals for the average difference between measurements between humans and the AI predictions. The left and right measurements were pooled for the bilateral measures. The mean absolute error between paired observations was calculated using the mean of the absolute value of paired differences between human and AI predicted measurements for each metric. The interrater reliability of the measurements was calculated using the intraclass correlation coefficient (ICC). The ICC estimates and 95% confidence intervals were calculated using the R package irr v0.84.1 based on single measures, absolute-agreement, 2-way mixed-effects model. ICC estimates were interpreted as poor reliability (ICC < 0.5), moderate reliability (0.5 < ICC < 0.75), good reliability (0.75 < ICC < 0.9), and excellent reliability (ICC > 0.90). Statistical analysis was performed using R v4.1.2. A p-value < 0.05 was considered statistically significant.

## Results

**Figure 3.**
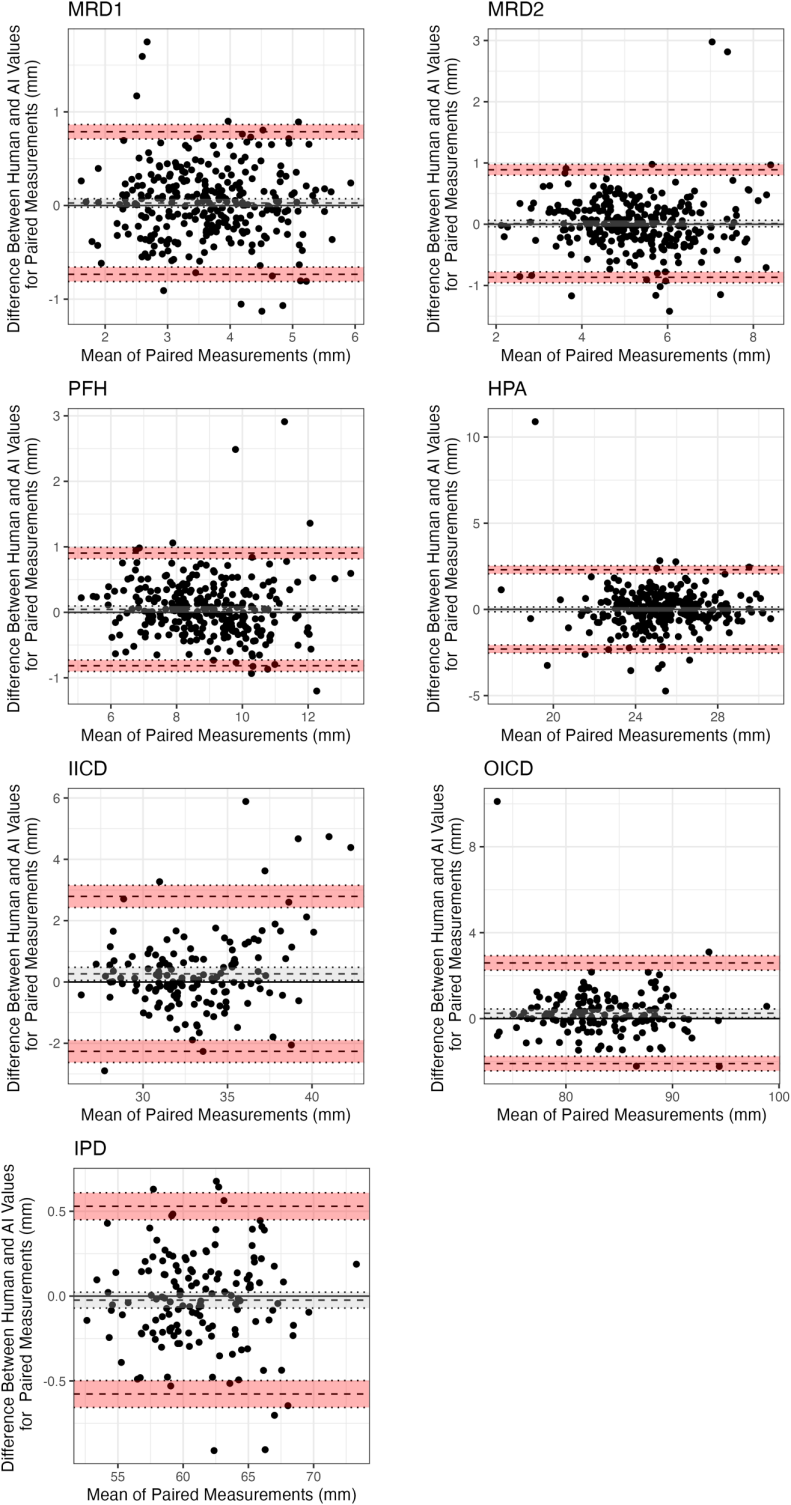
Bland-Altman plots demonstrating the bias and limits of agreement for periocular measurements. Bias (mean of differences) is the dashed dark grey line. Upper and lower confidence intervals of bias are depicted by the dotted grey lines and grey shading. Upper and lower limits of agreement are depicted by the dashed black lines. Their associated confidence intervals are depicted by the dotted black lines and red shading.

A total of 958 eyes were included from 479 participants. The mean age of participants was 59 ± 17.9 years and 257 (54%) were female. Most participants were Caucasian (407, 85%), with other groups being East Asian (34, 7.1%), South Asian (28, 6%), and African (10, 2.1%). The testing set consisted of 290 eyes from 145 patients. A summary and comparison of the human and AI predicted periorbital measurements are detailed in Table 1. On average, the AI predicted measurements were < 1 mm away from human measurements for all metrics (Table 2). The Bland-Altman plots are showed in Figure 3 with the bias and limits of agreement. The magnitude of difference between human and AI measurements was less for MRD1, MRD2, IPD and PFH (Figure 3). IICD showed a greater difference between measurements and less agreement for larger measurements (Figure 3). The intraclass correlation coefficients demonstrated excellent reliability for all measurements except HPA which showed good reliability (Table 3). The landmark detection model was highly accurate and achieved a mean error rate of 0.51% and failure rate at 0.1 of 0%.

**Table 1.** Comparison of mean periorbital measurements between human and AI predictions.

	Measured	Predicted
Metric	*N* = 290^1^	
MRD1	3.66 (0.89)	3.64 (0.90)
MRD2	5.24 (1.22)	5.23 (1.23)
PFH	8.90 (1.49)	8.86 (1.52)
HPA	25.30 (2.08)	25.30 (2.11)
	*N* = 145^1^	
IPD	61.2 (3.7)	61.2 (3.8)
OICD	83.7 (4.6)	83.5 (4.8)
IICD	33.3 (3.4)	33.0 (3.0)

^1^ Mean (SD).

MRD, marginal reflex distance; PFH, palpebral fissure height; HPA, horizontal palpebral aperture; IPD, interpupillary distance; OICD, outer intercanthal distance; IICD, inner intercanthal distance.

**Table 2.** Mean absolute error between paired measurements taken by human and AI predictions.

Metric	Mean Absolute error (standard deviation)
MRD1	0.29 (0.25)
MRD2	0.30 (0.32)
PFH	0.31 (0.31)
HPA	0.74 (0.91)
OICD	0.73 (0.97)
IICD	0.88 (0.97)
IPD	0.22 (0.18)

MRD, marginal reflex distance; PFH, palpebral fissure height; HPA, horizontal palpebral aperture; IPD, interpupillary distance; OICD, outer intercanthal distance; IICD, inner intercanthal distance.

**Table 3.** Intraclass correlation coefficients for measurement reliability for each periorbital metric.

Metric	ICC	95% CI	p
MRD1	0.906	0.883–0.925	<0.0001
MRD2	0.933	0.917–0.947	<0.0001
PFH	0.957	0.946–0.966	<0.0001
HPA	0.843	0.806–0.873	<0.0001
OICD	0.966	0.953–0.976	<0.0001
IICD	0.918	0.887–0.941	<0.0001
IPD	0.997	0.996–0.998	<0.0001

MRD, marginal reflex distance; PFH, palpebral fissure height; HPA, horizontal palpebral aperture; IPD, interpupillary distance; OICD, outer intercanthal distance; IICD, inner intercanthal distance.

## Discussion

We present the successful development of an automated facial landmark detection algorithm for periocular measurements. Our study demonstrates the potential to create a highly accurate landmark detection model which can automatically detect key periocular landmarks and use these to calculate periocular measurements. The algorithm produced results less than 1 mm from human measurements with excellent reliability. This automation has the potential to overcome the subjectivity and operator-dependent variability associated with manual measurements This AI-based approach can significantly improve the efficiency of periocular measurements and facilitate remote patient assessment, making it particularly relevant in the context of increasing telehealth use.

Previous studies have developed methods to calculate MRD1 and MRD2 with less human input. Bodnar, Neimkin^3^ utilised edge detection techniques, including the Canny edge detection method, to identify facial features and estimate MRD1 and MRD2, and Lou, Yang^4^ employed a facial landmark detection program in combination with edge detection to recognise the pupillary centre and estimate MRD1 and MRD2. Thomas, Gunasekera^5^ used OpenFace, an open-source AI driven facial analysis software, to measure the vertical palpebral aperture. However, this study did not calculate the MRD1 and MRD2 measurements specifically. Our methodology uses deep learning algorithms to detect periocular landmarks and calculate periocular dimensions, including but not limited to MRD1 and MRD2. Machine learning models provide increased robustness to variations found in real-world images such as lighting conditions, angles, facial size, and expressions. Additionally, deep learning techniques automatically learn features from the raw imaging data enabling accurate localisation of key periocular landmarks. In our study, we adopted the widely used HRNet-V2 as our backbone network to learn the high-resolution representations through the whole process for facial landmark detection. Traditional computer vision techniques can have difficulty detecting facial landmarks due to large head position, and heavy occlusion. By training a Convolutional neural network (CNN) on a dataset of images containing labelled facial landmarks, the algorithm can identify facial features in new images and achieve high detection performance in a variety of conditions.

The accurate conversion of pixels to millimetres in images is required, and different studies have adopted different techniques for this purpose. In the Van Brummen study, the AI algorithm's pixel-to-mm conversion relied on a corneal width of 11.71 mm, which was different from the corneal width of 11.77 mm measured by human graders.^6^ Moreover, measuring the corneal width can pose challenges, particularly in cases of ptosis where the eyelid's position may cover part of the cornea.^7,8^ In our study, we affixed a sticker dot with a known diameter on the forehead of each participant to allow a reliable and standardised conversion of pixels to mm. All participants had this performed as our study consisted of solely prospectively enrolled patients, in contrast to the Van Brummen study, which primarily relied on retrospective recruitment.^6^

Limitations to this study include the data being from a single centre, although the patients studied were diverse in terms of clinical presentation, age, sex, and ethnicity. This real-world sample size reflects the typical patient population seen in ophthalmology clinics, enhancing the generalisability of our findings to other Australian cohorts. A multicentre study would however have greater generalisability. Our AI system compared the measurements done by human graders on images taken in clinic. Comparison with manual measurements done in clinic would allow us to determine whether the AI system performed comparably as factors such as frontalis muscle action and subtle blinking may not be completely controlled for with a single image. In addition, the photos were taken in standardised conditions and need to be externally validated on images from different cameras and settings prior to being used in a Telehealth setting.

In conclusion, our study introduces an automated facial landmark detection network for periocular measurements, providing accurate and reliable results. The AI algorithm's successful development opens avenues for objective and efficient assessment of periocular structures. As telehealth becomes more prevalent, the implementation of such automated measurement techniques can streamline remote patient assessment and improve ophthalmic care delivery.

## Financial disclosures/declaration of conflicting interests

The authors declared no potential conflicts of interest with respect to the research, authorship, and/or publication of this article.
